# Optimizing Osseodensification Drilling for Dental Implant Placement: An In Vitro Study

**DOI:** 10.1002/cre2.70155

**Published:** 2025-06-11

**Authors:** Xingru Tao, Jie Yang, Tai Ma, Ming Chen, Qinglong An, Dedong Yu

**Affiliations:** ^1^ Department of Second Dental Center, Shanghai Ninth People's Hospital, College of Stomatology, School of Medicine Shanghai Jiao Tong University Shanghai China; ^2^ National Clinical Research Center for Oral Diseases Shanghai China; ^3^ Shanghai Key Laboratory of Stomatology & Shanghai Research Institute of Stomatology Shanghai China; ^4^ State Key Laboratory of Mechanical System and Vibration, School of Mechanical Engineering Shanghai Jiao Tong University Shanghai China; ^5^ Fengcheng Branch, Shanghai Ninth People's Hospital Affiliated to Shanghai Jiao Tong University School of Medicine Shanghai China

**Keywords:** dental implant, osseodensification drilling, polyurethane foam blocks, primary stability, Type IV bone

## Abstract

**Objectives:**

This study aims to optimize the parameters of osseodensification drilling (OD) to improve the primary stability of dental implants in low‐density bone.

**Material and Methods:**

Polyurethane foam blocks (PFB) of 0.160 g/cm^3^ were used to simulate low‐density bone (Type IV bone). Two drills, i.e., bone level tapered drills and Densah drills, were used in conventional drilling (CD) and OD, respectively. Drilling was performed on a DMU machine, varying spin speed, feed per tooth, and irrigation with or without 4°C saline. Thrust force, temperature, and entrance characteristics of implant sites in CD and OD were compared. The primary stability of implants was assessed via insertion torque (IT), removal torque (RT), and implant stability quotient (ISQ).

**Results:**

OD demonstrated higher thrust force than CD with maximum values at 300 rpm (2.99 ± 0.22 N vs. 2.77 ± 0.17 N, *p* < 0.01). Temperature elevation was lower in OD than that in CD under irrigation (3.35°C vs. 4.67°C). Despite comparable ISQ values (CD: 46.71 ± 8.56 vs OD: 47.08 ± 5.95, *p* = 0.86), OD achieved higher IT (11.73 ± 0.45 N·m vs. 7.77 ± 0.21 N·m, *p* < 0.001) and higher RT (9.28 ± 0.45 N·m vs. 6.65 ± 0.19 N·m, *p* < 0.001). Morphological analysis revealed fewer defects (tears/potholes) in OD than CD.

**Conclusions:**

OD drills may avoid iatrogenic damage of the morphology of implant sites and enhance primary stability in Type IV bone at 1500 rpm and 0.04 mm/z with irrigation to prevent thermal damage. OD also outweights CD in increased bone density, thrust force, and torque.

**Clinical Implications:**

For Type IV alveolar bone, OD at 1500 rpm and 0.04 mm/z is recommended for improving he primary stability of dental implants. Sufficient irrigation is crucial in both CD and OD for circumventing the thermal damage to bone.

## Summary

**What Is Known?:**

- Osseodensification drilling (OD) techniques can enhance the primary stability of dental implants in low‐density bone, but optimal drilling parameters remain unveiled.

**What This Study Adds?:**

- Identified optimized OD parameters (1500 rpm, 0.04 mm/z feed, irrigation) to provent the impaired implant sites and improve torque values (IT/RT) versus CD.

- Demonstrated OD increases bone density and thrust force while maintaining comparable ISQ, offering a reliable method for improving the primary stability of implants in Type IV bone.

**Abbreviations**
CD: conventional drilling
ISQ: implant stability quotient
IT: insertion torque
OD: osseodensification drilling
PFB: polyurethane foam blocks
RT: removal torque

## 1 Introduction

Dental implants have been predominantly utilized for oral rehabilitation in partial or complete edentulous cases owing to the pronounced long‐term success rate. Appropriate density of bone around inserted implants is vital for this long‐term success as it may significantly facilitate the firm and direct integration between bone and dental implants, i.e., osteointegration (Frisch et al. 2020; Chauhan et al. 2018; Kotsakis and Romanos 2022). For dental implant placement, the holes with expected diameters and depths in the jawbone should be prepared by drills. Conventional drilling (CD) techniques employ drills with sequential diameters to create cylindrical osteotomies or conical holes, and the diameter of the final drill is usually equal to the implants. However, in osteoporotic areas, the diameter of prepared sites is usually larger than that of the final drills and the implants, resulting in inadequate osteointegration and losing the primary stability (Lekholm UJTip 1985; Putra et al. 2024). To address this clinical challenge, some strategies were proposed. For instance, osteotome technique was introduced in cases with insufficient bone height and density for dental implant insertion in post‐maxilla areas (Summers 1994). It increases bone density by grafting a layer of bone or bone substitutes on the surface of residual bone, ultimately improving the primary stability of the inserted dental implant. However, this procedure may cause severe side effects, including unexpected trauma to the patients, accidental fractures, and bone displacement.

Alternatively, Huwais and Meyer first introduced a method, i.e., “osseodensification” drilling (OD), for preparing implant sites in bone with inferior density (Huwais and Meyer 2017). This technique can achieve lateral bone extrusion and increase bone density at the contact interface with the implant (Mello‐Machado et al. 2021; Tabassum et al. 2010; Bergamo et al. 2021), thereby finally increasing the primary stability of dental implants. In addition, OD reduces heat generation, diminishing the potential thermal damage to bone during the implant site preparation (Mullings et al. 2021; Bhargava et al. 2023; Yeh et al. 2021; Slete et al. 2018).

A wealth of studies have confirmed superior penetration forces, torque, and primary stability of dental implants after using OD than using CD techniques. Bettach et al. documented that OD in low‑density bone generated a significantly higher torque than CD (Bettach et al. 2023). As with increased torque, the improved primary stability of dental implants and more heat production were also reported. A systematic review revealed that a significant increase in the insertion torque (IT) of the implants positioned through the OD protocol compared to the CD (Inchingolo et al. 2021). Additionally, compared with the osteotome technique, OD is less invasive and less likely to induce fracture, thereby circumventing some complications, such as dizziness and infection.

However, Huwais and other related studies did not recommend exact parameters used in OD for clinical practice. Hence, this study is designed to explore the potentially optimal parameters, including thrust force, real‐time temperature around the bone during drilling, IT, and removal torque (RT) in OD, which may guide clinical practice.

## 2 Materials and Methods

**Figure 1:**
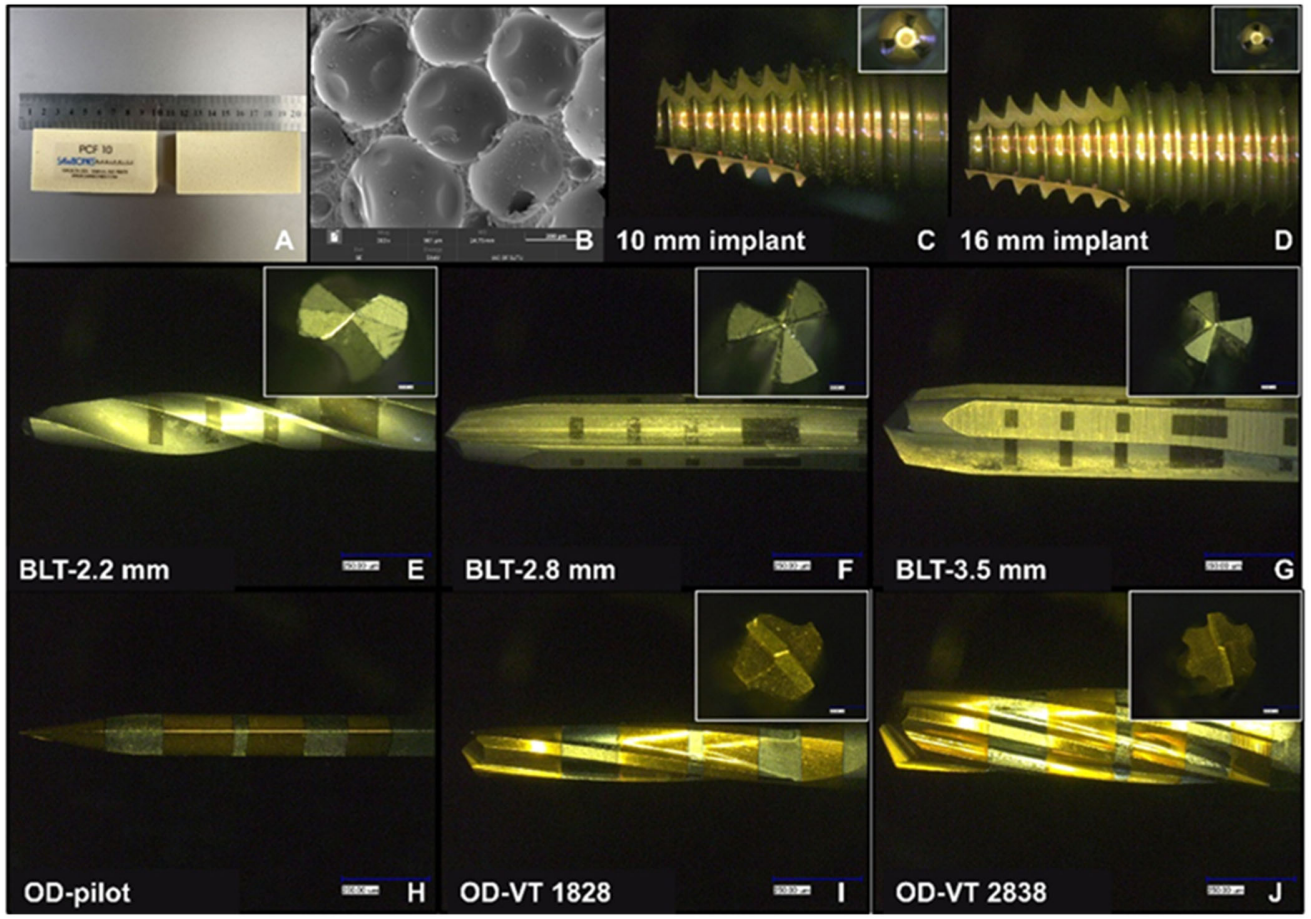
Involved materials in the study. (A) PFB; (B) SEM image of PFB; (C) 10 mm long implant; (D) 16 mm long implant; (E–G) bits of BLT drills; (H–J) bits of Densah drills.

In this study, polyurethane foam blocks (PFB) (Sawbones, Vashon Island, Washington, D.C., USA) of 0.160 g/cm^3^ (PCF 10) were used to simulate low‐density bone (Type IV bone), as standardized by the American Society for Testing and Materials (Standard Specification for Rigid Polyurethane Foam for Use as a Standard Material for Testing Orthopaedic Devices and Instruments n.d). The dimensions of the PFB used in the experiments were 80×40×20mm, and SEM images of the PFB material are shown in Figure 1B. BLT dental implants with 4.1 mm diameter and 10 and 16 mm length (BLT, Straumann, Switzerland) were used in this study (Figure 1C,D). Saline was stored in 4°C for irrigation during drilling. Ethics approval was waived for this In Vitro study.

Based on a previous study that used 40 samples of PFB models (de Carvalho Formiga et al. 2021), we calculated the sample size using G*Power (Standard Edition, Universität Düsseldorf, Düsseldorf, Germany). After a comprehensive consideration of various factors, 48 samples were recruited in this experiment (effect size = 0.92, α = 0.05, power = 0.8). Osteotomies were created in the PFBs using two different drills: bone‐level tapered (BLT) drills for CD (BLT, Straumann, Switzerland) and Densah drills (Versah, Jackson, Michigan, USA) for OD. The BLT drills contains three different drill bits: a 2.2 mm diameter pilot drill, 2.8 and 3.5 mm diameter drills. Three Densah drills were employed in the present study: a 1.6 mm diameter pilot drill bit, 1828 (cone shape; 18 means the secondary 1.8 mm diameter; 28 means the main diameter of the crown is 2.8 mm), and 2838 drills. The materials used in this study are shown in Figure 1.

Osteotomy experiments were performed using a DMU computer numerical control machine. Three levels of spin speeds (N), two levels of feed per tooth ($F_{z}$), and two levels of irrigation were set (Table 1). According to Versah protocols, we used three spin speed: 300, 800, and 1500 rpm in this study. A drilling force measurement system (KISTLER 9272 dynamometer, Kistler Group, Switzerland) was used to measure the dynamic thrust force.

**Table 1:** Experimental parameters in this study.

Material	Spin speed (N) (rpm)	Feed per tooth Fz (mm/z)	Implant depth (mm)	Irrigation
PCF 10	300, 800, 1500	0.04, 0.08	10, 16	without saline; saline

**Figure 2:**
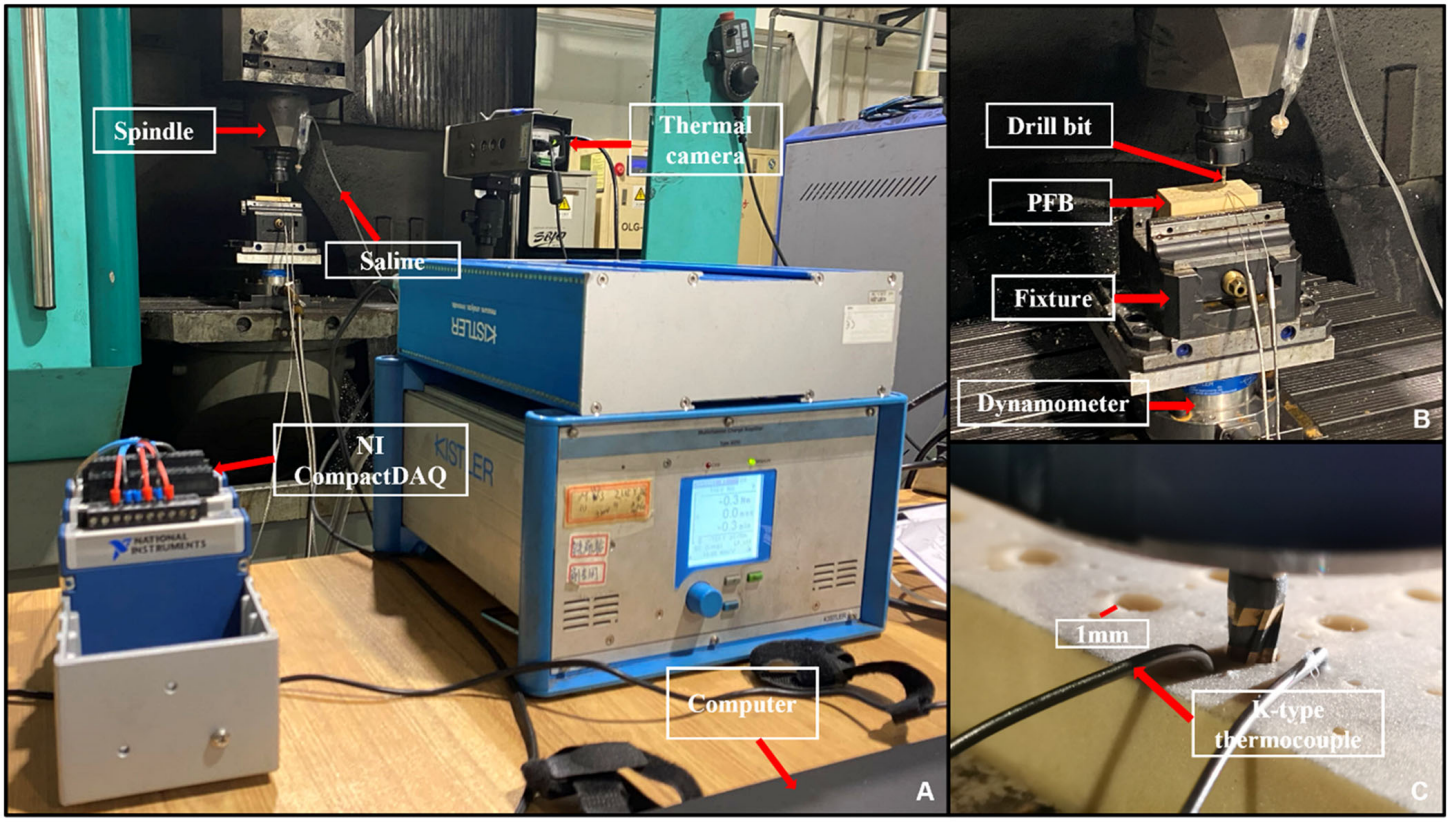
Drilling experimental setup. (A) machining center; (B) drilling platform; (C) schematic showing thermocouple distribution.

To collect the internal temperature of the material, a K‐type thermocouple was embedded in two diameter holes (Φ 1 mm) to collect the real‐time temperature at depths of 5, 10, and 16 mm (Figure 2C). Therefore, a thermocouple temperature‐measuring system was designed using NI LabView to analyze the temperature inside the hole. The surface temperatures of the PFB and drills were measured using an infrared thermal imager (FLIR A615, Teledyne FLIR, Wilsonville, OR, USA). The hole surface was evaluated using a material‐type upright laser confocal microscope (VK‐X3000, KEYENCE, Osaka, Japan) and a Raman image scanning electron microscope (RISE‐MAGNA, Magna, Canada). The IT and RT were measured using a torque‐testing machine (Tohnichi Mfg, Tohnichi, Japan), and the implant stability quotient (ISQ) was measured using a resonance frequency analysis system (Osstell, Gothenburg, Sweden). All involved instruments in this study are illustrated in Figure 2.

## 3 Statistical Analysis

SPSS 26.0 (IBM, Armonk, NY, USA) was used for the statistical analysis. Shapiro–Wilk test was used to conduct data normality analysis, and independent samples *t*‐test was used to compare differences between these two groups. All data were shown as mean ± standard deviation, and a statistically significant difference was set as *p* < 0.05.

## 4 Results

### 4.1 IT, RT, and ISQ Values

The primary stability of inserted implants in the two groups was evaluated by immediate IT, RT, and ISQ after their successful placement in the PFBs, and the details are listed in Table 2. Accodring to the Shapiro–Wilk test analysis results, the data are normally distributed. As shown in Table 2, the IT values in the OD group significantly increased. The IT values in CD and OD groups are 7.77 ± 0.21 and 11.73 ± 0.45, respectively, with a significant difference (*p* < 0.001). Likewise, the OD group has a higher RT value than the CD group (9.28 ± 0.45 and 6.65 ± 0.19, respectively), demonstrating a significant difference (*p* < 0.001). However, ISQ values in the four directions (buccal, lingual, mesial, and distal) show no significant differences between the CD and OD groups. The mean ISQ values for the CD and OD groups were 46.71 versus 47.08 (buccal), 45.67 versus 46.96 (lingual), 49.08 versus 49.71 (mesial), and 48.17 versus 49.92 (distal). The OD group consistently showed a slightly higher average ISQ value compared to the CD group.

**Table 2:** IT, RT, and ISQ in CD and OD groups.

Groups	Min. (N·m)	Max. (N·m)	SD	Average (N·m)	95%CI	S‐W test	*p*‐value
IT							
CD	5.80	9.50	1.04	7.77	7.33, 8.21	0.61	*p* < 0.001
OD	8.00	14.80	2.22	11.73	10.79, 12.67	0.05	
RT							
CD	4.80	9.20	0.92	6.65	6.26, 7.04	0.20	*p* < 0.001
OD	6.00	14.10	2.22	9.28	8.35, 10.22	0.26	
ISQ							
BLT‐buccal	28.00	64.00	8.56	46.71	43.10, 50.32	0.13	0.86
OD‐buccal	35.00	57.00	5.95	47.08	44.57, 49.60	0.19	
BLT‐lingual	21.00	65.00	9.39	45.67	41.70, 49.63	0.07	0.55
OD‐lingual	35.00	57.00	4.95	46.96	44.87, 49.05	0.32	
BLT‐mesial	25.00	65.00	9.32	49.08	45.15, 53.02	0.42	0.66
OD‐mesial	42.00	63.00	5.86	49.71	47.23, 52.18	0.09	
BLT‐distal	28.00	65.00	8.74	48.17	44.48, 51.86	0.17	0.22
OD‐distal	42.00	63.00	5.83	49.92	47.45‐52.38	0.143	

Abbreviations: CD, conventional drilling; IT, insertion torque; ISQ, implant stability quotient; OD, osseodensification; RT, removal torque.

### 4.2 Entrance Characteristics of Prepared Implant Sites

**Figure 3:**
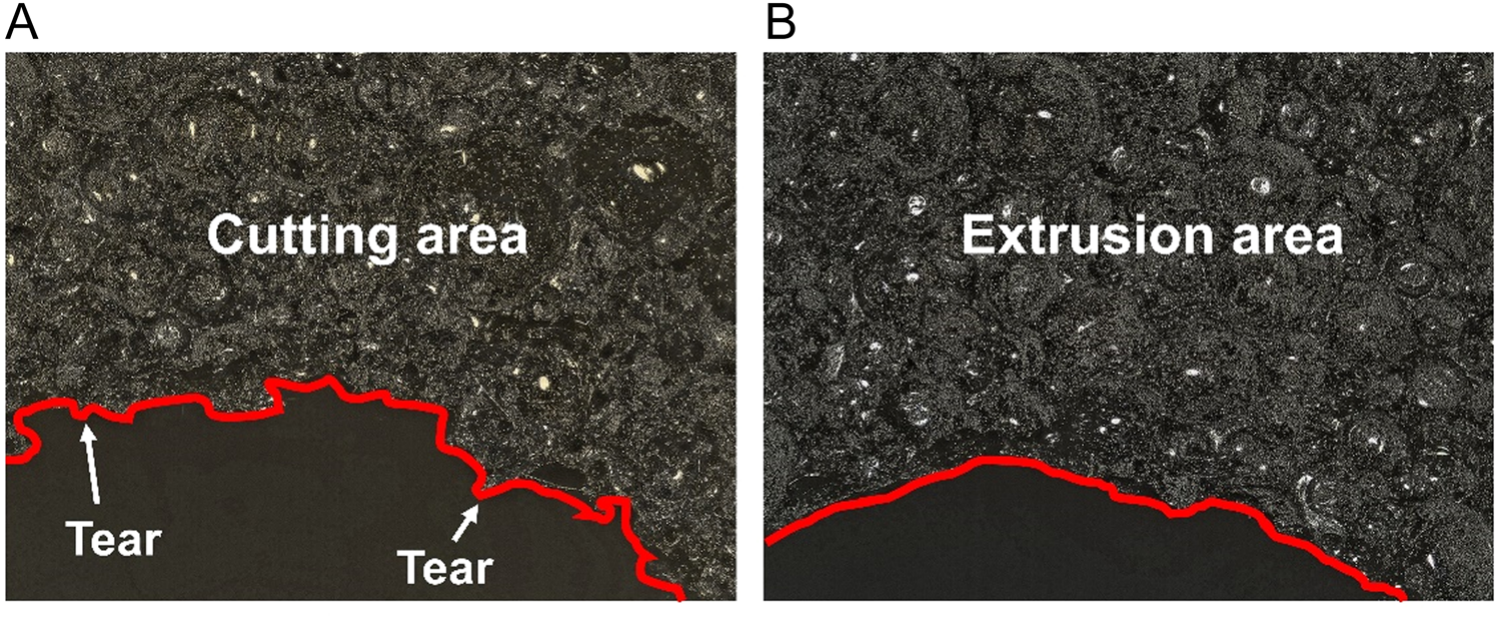
Characteristics of prepared implant sites. (A) implant sites prepared via BLT drills; (B) implant sites prepared via Densah drills.

The entrance characteristics of implant sites were assessed using a material‐type upright laser confocal microscope. In Figure 3, the entrance characteristics were varied when using different drills to prepare the implant sites under the same condition (N = 300 r/min, $F_{z}$ = 0.08 mm/z, depth = 10 mm, without irrigation). In the CD group, burrs, tears, and potholes were observed at the prepared sites. However, only a few burrs appeared in the OD group. During clockwise drilling, the cutting action of the tool generated burrs on the prepared surface. When the implant was inserted, burrs were further displaced due to torque, leading to tearing or the formation of depressions. In the counterclockwise mode, the tool compresses the material, causing localized fractures and adhesion of material to the prepared site walls. This compression increased the material's resistance to deformation during implant insertion, resulting in fewer occurrences of tearing or potholes, and only some burrs.

### 4.3 Spin Speed and Thrust Force

**Figure 4:**
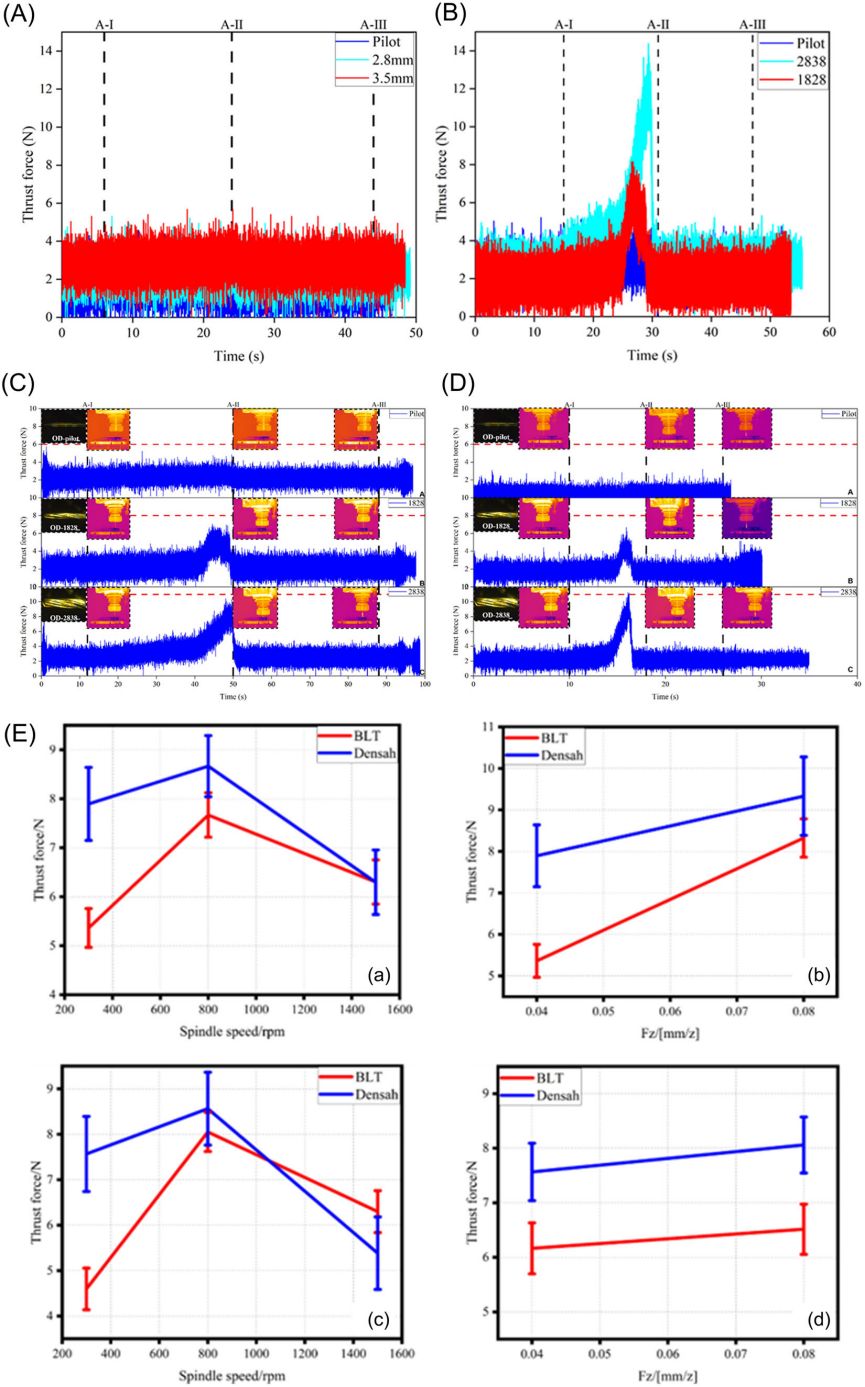
(A) Thrust force signals showing the bone level tapered (BLT) drills under drilling without irrigation during clinical routine method: Pilot, 2.8 mm, and 3.5 mm drills (drilling parameters: *N* = 300 r/min; *F*_z_ = 0.08 mm/z; implant hole depth = 16 mm). (B) Thrust force signals showing the Densah drills under drilling without irrigation during osseodensification method: Pilot, 1828, and 2838 drills (drilling parameters: *N* = 300 r/min; *F*_z_ = 0.08 mm/z; implant hole depth = 16 mm). (C) Thrust force signals showing the Densah drills under drilling without irrigation during osseodensification method: Pilot, 1828, and 2838 drills (drilling parameters: *N* = 300 r/min; *F*_z_ = 0.04 mm/z; implant hole depth = 16 mm). (D) Thrust force signals showing the Densah drills under drilling without irrigation during osseodensification method: Pilot, 1828, and 2838 drills (drilling parameters: *N* = 1500 r/min; *F*_z_ = 0.04 mm/z; implant hole depth = 16 mm). (E) Effect of spin speed and feed per tooth on the thrust force under drilling without irrigation (implant depth = 10 mm and 16 mm). E‐a: 10 mm implant, BLT and Densah groups, thrust force and spin speed (N from 300 to 1500 r/min); E‐b: 10 mm implant, BLT and Densah groups, thrust force and feed per tooth (F_z_ from 0.04 to 0.08 mm/z; E‐c: 16 mm implant, BLT and Densah groups, thrust force and spin speed (N from 300 to 1500 r/min); E‐d: 16 mm implant, BLT and Densah groups, thrust force and feed per tooth (F_z_ from 0.04 to 0.08 mm/z).

During drilling, the thrust force (in the axial direction) reflects different material machining stages. Figure 4a shows the thrust force of BLT drills under dry drilling during the following routine clinical procedures. With this method, the osteotomy is generally performed using bits of three different diameters (2.2, 2.8, and 3.5 mm). A 2.2 mm pilot drill bit was used to form a pre‐hole. The 2.8 and 3.5 mm drill bits were used to form the final implant hole to complete the osteotomy. The A‐I dashed line indicates the critical state when the pilot drill bit is ready to enter the PFB. The A‐II dashed line indicates the state at which the drill bit reaches the bottom of the PFB. Moreover, A‐Ⅲ represented the state of the drill bit as it exited the PFB. The thrust force produced by the BLT drill bits was relatively small, in the range of 0–6 N. The thrust force was 1.53 (pilot drill bit), 2.22 (2.8 mm drill bit), and 2.77 N (3.5 mm drill bit), respectively. However, using the same drilling parameters, the thrust force of the Densah drill bits is higher than that of the BTL drill bits (Figure 4b). The thrust force of the 1.6 mm pilot drill bit was 0.97–1.42 N (49.20–85.49%) higher than that of 2.2 mm pilot drill bit under the clinical routine method. The working mode of the pilot drill bit was clockwise cutting. The 2.2 mm BLT drill bit was built with more spiral grooves than the 1.6 mm Densah drill bit. As shown in Figure 4b, the thrust force has an evident tendency to increase. The thrust force of OD VT‐1828 and OD VT‐2838 was 0.32–0.67 N (21.10–61.58%) and 0.18–4.44 N (7.64–134.86%) higher than that of the 2.8 and 3.5 mm BLT drill bits, respectively. For drilling parameters: N = 300 r/min; $F_{z}\;$= 0.04 mm/z; implant depth: 16 mm. The thrust force produced by the BLT drill bits was 0–6 N. The thrust force was 1.53 (pilot drill bit), 2.28 (2.8 mm drill bit), and 2.65 N (3.5 mm drill bit). All three drill bits were rotated clockwise. Subtractive manufacturing has also been used. Force analysis of the PFB was conducted according to the theory of the three deformation zones. Because of the structural characteristics of the material, the cutting forces in the three deformation zones were relatively small. Therefore, the thrust force from the deformation zones was small.

However, the thrust force of Densah drill bits was 2.24 (pilot drill bit), 2.34 (OD VT‐1828), and 2.99 N (OD VT‐2838) (Figure 4c). In the osseodensification method, the tool is changed from clockwise to counterclockwise, and the material is squeezed by the tool instead of cutting. The thrust force increased by 46.41, 2.63, and 12.83%, respectively. For drilling parameters: N = 1500 r/min, $F_{z}$ = 0.04 mm/z; Implant depth = 16 mm. Thrust force was 0.44 (2.2 mm drill bit), 1.86 (2.8 mm drill bit), and 2.46 N (3.5 mm drill bit), respectively (Figure 4d). With an increase in the spin speed, the extrusion between the tool and material was reduced. Therefore, the thrust forces were reduced by 80.36, 20.51, and 17.73%, respectively.

The analysis in Figure 4e indicates that the thrust force initially increases and then decreases with rising spin speed (300–1500 r/min), for both BLT and Densah drill bits. At 300 r/min, the thrust force of Densah drill bits was approximately 47.22% higher than that of BLT drill bits, while at 800 r/min, it was 13.00% higher. At 1500 r/min, the thrust force for BLT drill bits was marginally higher (0.08%) than for Densah drill bits. The thrust force increased with feed per tooth for both BLT and Densah drill bits. At a 10 mm implant depth, the thrust force for BLT drill bits increased by 47.21% and 12.12%, while for Densah drill bits, the increase was approximately 22.73% and 23.72%, respectively.

### 4.4 Temperature

**Figure 5:**
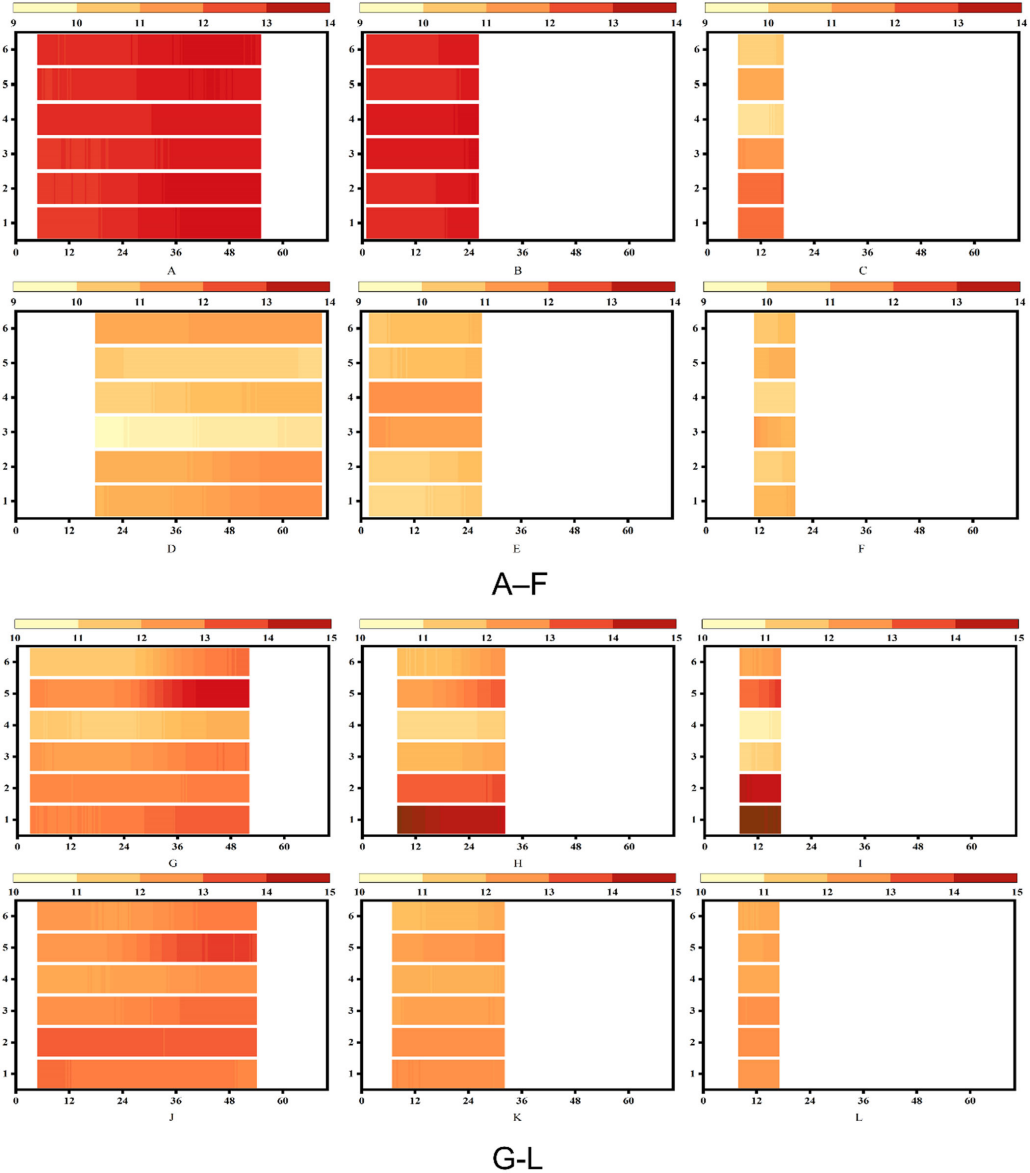
(A–F) Heat map showing the hole temperature under bone‐level tapered (BLT) drill bits (Implant depth: 10 mm; color level: 9–14). (G–L) Heat map showing the hole temperature under Densah drill bits (implant depth = 10 mm; color level: 10–15). The “1” and “2” on the vertical axis represent the pilot drill of BLT, “3” and “4” represent the 2.8 mm drill bit of BLT, and “5” and “6” the 3.5 mm drill bit of BLT. The odd number represented 5 mm depth, and the even number represented 10 mm depth.

**Figure 6:**
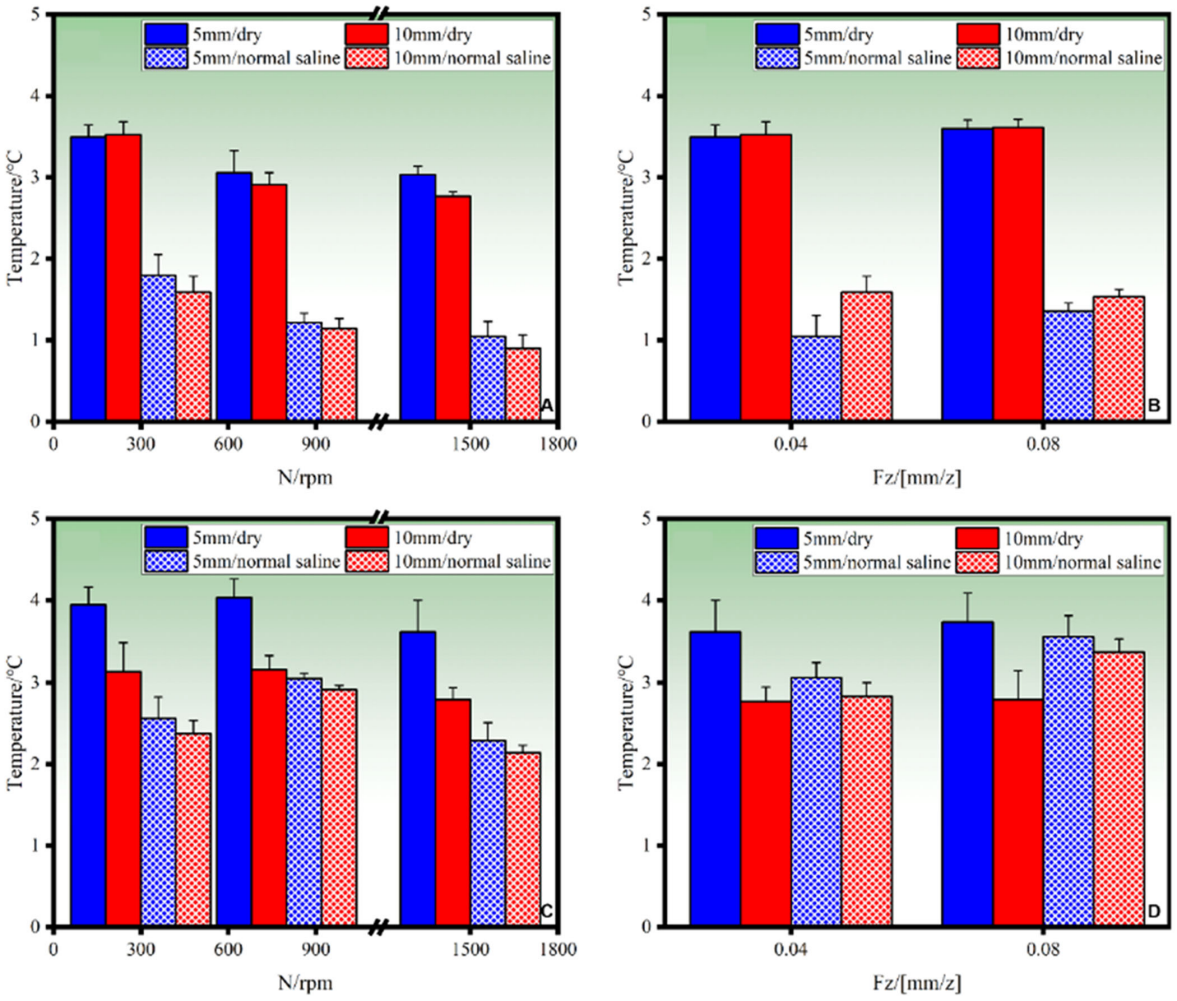
Effect of spin speed and feed per tooth on the $∆T_{\max}$ under drilling without irrigation: (A, B) bone‐level tapered BLT drill bits; (C, D) Densah drill bits; implant depth: 10 mm.

The temperature data collected in various intervals, i.e., 0–70 s, were used to analyze the heat product affected by different machining parameters. Figure 5A,B show the temperature changes under certain drilling parameters (300 rpm, 0.04 mm/z and 1500 rpm, 0.04 mm/z); Without irrigation, the average maximum temperature ($T_{\max}$) of BLT drilling reached 12.66°C at 300 rpm and 0.04 mm/z; at 300 rpm and 0.08 mm/z, the $T_{\max}$ slightly increased to 12.71°C. Conversely, at 1500 rpm and 0.04 mm/z, $T_{\max}$ increased to 10.91°C, demonstrating a 13.82% reduction attributed to decreased friction at higher spin speeds. During drilling with irrigation, as shown in Figure 6C, the $T_{\max}$ reached 10.67°C under 300 rpm and 0.04 mm/z, while under 300 rpm, 0.08 mm/z and 1500 rpm, 0.04 mm/z, the $T_{\max}$ reached 11.57°C and 10.39°C, respectively, reflecting the cooling effect of irrigation. The same trend was observed with increasing feed per tooth and spin speeds. Notably, irrigation reduced $T_{\max}$ by 15.72% (12.66°C–10.67°C) at 300 rpm and 0.04 mm/z, and by 8.97% (12.71°C–11.57°C) at 300 rpm and 0.08 mm/z.

For Densah drills, Figure 5G–L illustrates the same trends. Under drilling without irrigation, $T_{\max}$ was 12.74°C at 300 rpm and 0.04 mm/z, slightly increasing to 12.81°C at 300 rpm and 0.08 mm/z. However, at 1500 rpm and 0.04 mm/z, $T_{\max}$decreased to 12.62°C. This trend suggests that while higher feed rates slightly increase the heat due to thicker chips, higher spin speeds reduce friction, resulting in lower $T_{\max}$. With irrigation, the $T_{\max}$ reached 12.68, 12.07, and 12.05°C, respectively. The $T_{\max}$ for drilling with saline showed 5.26 (from 12.74°C to 12.68°C), 5.78 (from 12.81°C to 12.07°C), and 4.52% (from 12.62°C to 12.05°C) decreased. The use of saline effectively reduced the heat; however, less temperature decrease in the OD group was identified.

Figure 6 highlights temperature differences ($∆T_{\max}$) at various depths. For BLT drills, at a depth of 5 mm under drilling without irrigation, $∆T_{\max}$ decreased by 3.52% with increasing spin speed, attributed to reduced tool‐material friction. A similar trend was observed at 10 mm depth. Under irrigation, at 5 mm depth, $∆T_{\max}$ decreased by 13.59%, 15.22%, and 16.49%, with the maximum reduction of 21.12% observed at 10 mm depth (from 3.5°C to 0.90°C). However, increasing feed per tooth slightly increased $∆T_{\max}$, attributed to the greater material thickness removed per revolution. For Densah drills, $∆T_{\max}$ followed a different trend under drilling without irrigation. Initially, it increased with spin speed due to extrusion friction, rising by 0.70% (from 3.94°C to 4.03°C at 5 mm depth). Beyond a threshold speed, $∆T_{\max}$decreased by 3.07% (from 4.02°C to 3.62°C), as reduced contact time lessened the extrusion effect. Under irrigation, $∆T_{\max}$ decreased by 14.51% (from 4.03°C to 2.14°C) at 5 mm depth. While the trend of temperature reduction with increasing spin speed was similar to BLT drills, the decrease for Densah drills was less significant.

As summarized in Table 3, both BLT and Densah drills benefited from saline irrigation in reducing thermal damage during dental implant site preparation. However, BLT drills showed greater temperature reduction efficiency compared with Densah drills. This highlights the importance of optimizing drilling parameters and irrigation methods to minimize thermal damage and improve clinical outcomes.

**Table 3:** The ∆Tmax during the implant site preparation with 16 mm depth.

No.	Drill bit	Spin speed N (r/min)	Feed per tooth Fz (mm/z)	Cold fluid	∆Tmax(°C)
1	BLT	300	0.04	Without irrigation	5.13
2	BLT	300	0.08	Without irrigation	6.00
3	BLT	800	0.04	Without irrigation	5.01
4	BLT	800	0.08	Without irrigation	5.52
5	BLT	1500	0.04	Without irrigation	4.65
6	BLT	1500	0.08	Without irrigation	4.67
7	BLT	300	0.04	Saline	3.43
8	BLT	300	0.08	Saline	3.64
9	BLT	800	0.04	Saline	3.19
10	BLT	800	0.08	Saline	3.44
11	BLT	1500	0.04	Saline	3.19
12	BLT	1500	0.08	Saline	3.25
13	Densah	300	0.04	Without irrigation	4.46
14	Densah	300	0.08	Without irrigation	4.78
15	Densah	800	0.04	Without irrigation	4.80
16	Densah	800	0.08	Without irrigation	4.88
17	Densah	1500	0.04	Without irrigation	3.69
18	Densah	1500	0.08	Without irrigation	4.34
19	Densah	300	0.04	Saline	3.84
20	Densah	300	0.08	Saline	3.93
21	Densah	800	0.04	Saline	3.84
22	Densah	800	0.08	Saline	4.32
23	Densah	1500	0.04	Saline	3.35
24	Densah	1500	0.08	Saline	3.85

### 4.5 Optimized Drilling Parameters

Comparative analysis revealed distinct mechanical differences between OD and CD. The OD protocol generated elevated thrust forces and cutting resistance during cortical bone preparation, accompanied by increased thermal generation. However, this technique demonstrated superior performance in implant stability metrics, showing significantly enhanced insertion and removal torque compared to CD.

**Figure 7:**
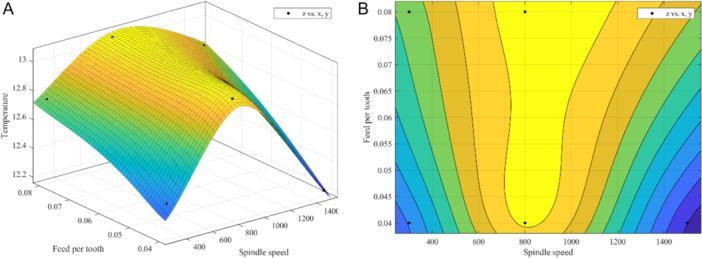
(A) Three‐dimensional (3D) image showing the effect of spin drilling speed and feed per tooth on temperature. (B) Contour map showing the effect of spin drilling speed and feed per tooth on temperature.

A three‐dimensional function involving speed, feed per tooth, and heat production was established in this study to explore the optimal parameters, aiming to identify the optimal parameters that minimize heat production during drilling. Based on the results, this study suggested a set of parameters could be used in clinical OD performance: the spin speed and feed per tooth were set as 1500 rpm and 0.04 mm/z, respectively (Figure 7).

## 5 Conclusion

OD can improve thrust force, IT, and RT during and after implant site preparation in light of more compacted surrounding bone compared with CD. The potential optimal spin speed and feed rate per tooth in OD are 1500 rpm and 0.04, respectively. In addition, irrigation is vital to eschew thermal injury to bone during drilling in both CD and OD.

## 6 Discussion

After the first introduction of “osseodensification” by Huwais and Meyer in 2017, this strategy has been receiving increasing attention when preparing implant sites in bone with compromised density. Meanwhile, exploring the optimal parameters for OD to improve the clinical outcomes in implant dentistry remains ongoing.

Regarding the primary stability of implants, our data align with previous studies that OD leads to better primary stability than CD (Kalra et al. 2024; Fontes Pereira et al. 2023). To evaluate the primary stability of implants, besides IT, the most used parameter, RT that is rarely used in the clinical due to the ethical consideration can also quantify the mechanical interlock strength at the bone‐implant interface as a secondary assessment metric In Vitro (Orhan and Ciğerim 2024; Kim et al. 2020). According to our study, OD, compared with CD, significantly enhances the primary stabilily of implants, validated by increased IT and RT. Although this result agrees with the data from Kalra et al. we offer a different interpretation of the underlying mechanism (Kalra et al. 2024). Kalra et al. suggest that elevated primary and secondary stability are attributed to bone densification along the osteotomy walls and the presence of residual bone chips when using OD, while we suspect the differences in diameter between OD and CD drills may explain this phenomenon. The interfacial frictional forces at the implant‐osteotomy interface exhibit diameter‐dependent variations: larger implant diameters generate significantly greater bone thread engagement resistance, thereby directly modulating the biomechanical coupling efficacy, which is critical for primary stability establishment. The average diameter of CD drills in the present study is 3.5 mm; however, based on the diameter calculation, the diameter of 2838 drill at 10 mm is 3.44 mm.

Clinically, it is generally recommended that the initial IT of an implant should be more than 35 N·m and the value of ISQ should be above 70 in. immediate loading cases. Research indicated that there is a positive correlation between IT and initial ISQ (Park et al. 2012). However, excessive IT during implant placement in dense bone can contribute to implant fracture. Exceed IT may also induce other adverse events. Alqahtani et al. documented that in D4 bone (serverly osteoporotic bone), implants with varying diameters and thread configurations were also associated with abnormal stress levels under immediate loading conditions (Alqahtani et al. 2023). It has been well‐acknowledged that in temperatures over 53°C lasting more than 1 min, the viability of various cells in bone is significantly impaired (Eriksson et al.^1982^). The temperature during drilling is affected by some factors, such as the bone type, drill diameters, spin speed, and irrigation (Vaidya et al. 2023; Soldatos et al. 2022; Chen et al. 2021; Gaspar et al. 2013; Gehrke et al. 2013; Salomó‐Coll et al. 2021; Chen et al. 2019; Strbac et al. 2014; Alam et al. 2023). In our study, the temperatures during CD and OD were both below 53°C; however, the profiles of temperature changes in CD and OD groups are different. The temperature during CD consistently decreased with increasing spin speed. As the spin speed and material removal rate increase, the contact between the drill and the material decreases, resulting in reduced friction and the heat generation. By contrast, in OD, the temperature rose and then fell with increasing spin speed. Intringuingly, in Huwais and Meyer's study, the temperature in OD consistently increased and was higher than CD. We assume this discrepancy is linked to the higher spin speed used in our study. In OD, the drill rotated counterclockwise, and the material was crushed and broken, subsequently adhering to the wall of the hole. At low spin drilling speed, the squeezing between the tool and material was gentle. Nonetheless, with increased spin speed, the extrusion friction between the tool and material surged. Of note, when the spin speed exceeded a certain threshold, the contact time between the tool and the material decreased, and the extrusion friction effect became small accordingly.

The temperature was also affected by irrigation. Irrigation can drastically decrease bone temperature during drilling, and a low‐temperature irrigation media can further reduce heat generation during implant site preparation (Saxena et al. 2024; Bulloch et al. 2012; Boa et al. 2016). In our study, external irrigation was employed, significantly decreasing temperature during OD and CD, which was not investigated in Huwais's study. Comparative studies on these irrigation techniques have been conducted. A study by Srtbac et al. found that combined irrigation can provide sufficient reduction in temperature changes during CD. However, the optimal irrigation method for OD remains under exploration (Strbac et al. 2014). In this study, we did not evaluate the effect of these two irrigation modes on temperature decreasement in OD. In future, a more comprehensive research will be scheduled to provide detailed information on this parameter and optimize the irrigation practice in clinical in OD.

Moreover, the characteristics of the dental implant site preparation varied from OD to CD in our study. It was discovered that only the powdery debris attached to the hole wall were identified in OD groups. In contrast, entrance morphology of prepared sites in CD groups features burrs, tearing, and potholes in the clockwise cutting mode. Studies demonstrated that implant designs affected osseointegration (Causey et al. 2021). Sergio et al. suggested that implants with different macrogeometries affect the IT and ISQ (Gehrke et al. 2023). However, the feature of the hole entrance morphology was not reported in studies before. However, our experiments did not prove that burrs have the same effect on animal bones. This result encourages us to schedule further experiments to explore whether the surrounding morphology affects further osseointegration or whether the powdery debris and burres around the entrance improve implant success.

The PFB used in the study may also be responsible for distinguishing results, as Huwais and Meyer prepared implant sites in porcine tibial plateau bones. Although this PFB can simulate Type IV bone, the density of actual bone is heterogeneous due to the distinct distance from the cortical bone to the bone marrow cavity. Polyurethane blocks with homogeneous density provide reproducibility and control, making them suitable for In Vitro comparisons. This study provided practicable parametric guidance for different osteotomies of human Type IV bone. Besides, polyurethane foam models have mechanical properties similar to bone tissue, and the mechanical properties allow the standardization of procedures by eliminating existing anatomical and structural differences in bone (Gehrke et al. 2018; Romanos et al. 2018). Thus, it can serve as a bone substitute In Vitro alternatives from a biomechanical perspective. Bano et al. revealed that polyurethane foam can be preferred as a bone substitute in biomechanical studies for it can estimate strain in the peri‐implant region (Bano and Shaukat 2025). Our findings on the thrust force, temperature, implant stability, IT and RT, and hole entrance morphology shed light on the clinical practice. While polyurethane foam models offer standardized testing platforms, their inherent material characteristics fundamentally differ from the biomechanical behavior of human bone. The synthetic substances' homogeneous structure and static viscoelastic properties fail to replicate the anisotropic architecture and spring‐back effect inherent to osseous tissue, potentially introducing deviations in implant stability assessments that may not fully correlate with clinical scenarios. In addition, polyurethane foam models cannot provide histological evidence. In the future, to collect stronger evidence, we plan to conduct animal studies, using cone‐beam computed tomography on animal bones before the experiment to ensure consistent bone density conditions. For instance, a study conducted by Frizzera et al. used pig mandibles to evaluate osseodensification, providing valuable insights into bone behavior during implant procedures (Frizzera et al. 2022).

## Author Contributions

**Xingru Tao:** writing – original draft, writing – review and editing, methodology, conceptualization, visualization. **Jie Yang:** writing – original draft, writing – review and editing, visualization, formal analysis. **Tai Ma:** investigation. **Ming Chen:** supervision, project administration. **Qinglong An:** writing – review and editing, resources, supervision, conceptualization. **Dedong Yu:** resources, supervision, funding acquisition.

## Ethics Statement

The authors have nothing to report.

## Consent

The authors have nothing to report.

## Conflicts of Interest

The authors declare no conflicts of interest.

## Data Availability

The data supporting the findings of this study are available from the corresponding author upon reasonable request.
