# Correction to “Optimizing Osseodensification Drilling for Dental Implant Placement: An In Vitro Study”

**DOI:** 10.1002/cre2.70176

**Published:** 2025-07-24

1

Tao, X., Yang, J., Ma, T., Chen, M., An, Q., & Yu, D. (2025). Optimizing Osseodensification Drilling for Dental Implant Placement: An In Vitro Study. *Clinical and Experimental Dental Research* 11(3), e70155. https://doi.org/10.1002/cre2.70155

In the affiliation section of the published article, the co‐corresponding author Dr. Qinglong An was incorrectly listed under:

“5 Fengcheng Branch, Shanghai Ninth People's Hospital Affiliated to Shanghai Jiao Tong University School of Medicine, Shanghai, China”

Correction:

The correct affiliation for Dr. Qinglong An is:“4 State Key Laboratory of Mechanical System and Vibration, School of Mechanical Engineering, Shanghai Jiao Tong University, Shanghai, China”

We apologize for this error.

